# Effective new membrane for preventing postthoracotomy pleural adhesion by surface water induction technology

**DOI:** 10.1371/journal.pone.0179815

**Published:** 2017-06-27

**Authors:** Akiko Uemura, Mary Nakata, Seijirow Goya, Toshiharu Fukayama, Ryou Tanaka

**Affiliations:** 1 Tokyo University of Agriculture and Technology Animal Medical Center, Fuchu-shi, Tokyo, Japan; 2 National Cerebral and Cardiovascular Center, Suita, Osaka, Japan; Peking University People's Hospital, CHINA

## Abstract

**Background:**

After thoracic surgery, adhesions between the pleura can cause substantial complications. This study investigated the effectiveness of a novel membrane utilizing surface water induction technology to prevent adhesions.

**Methods:**

Eight beagles were divided into an experimental group (five males) and a control group (three females). The experimental group underwent thoracotomy on both the left and right sides of the chest. Both sides received the membrane, and the membrane on one side was glued to the pleura using tissue adhesive. The control group underwent thoracotomy only on the left side. Two weeks postoperatively, all dogs were sacrificed and adhesions were evaluated macroscopically and microscopically.

**Results:**

Severe adhesion was seen between the parietal and visceral pleura in all control dogs, whereas the experimental group showed minor adhesion in only one dog on one side.

**Conclusions:**

Our novel anti-adhesive membrane appeared highly effective in preventing postthoracotomy pleural adhesions.

## Introduction

Postoperative adhesions occur at a high rate following surgical operations. In thoracic surgery, these adhesions prevent normal re-expansion of the lungs and thus interfere with respiratory function [1], as well as protracting the time until subsequent surgery can be performed, increasing the risk of hemorrhage, restricting the field of view, and damaging pulmonary blood vessels [2–4].

In Japan, a total of 67,325 cardiovascular operations were performed in 2013, along with 75,306 general thoracic surgeries [5], compared with 903,813 abdominal operations (based on the 2013 Diagnosis Procedure Combination data distributed by the Ministry of Health, Labour and Welfare). Preventing postoperative adhesions has long been the subject of research [6–9], and a membrane for preventing abdominal postoperative adhesions has already been developed and commercialized under the brand name Seprafilm^®^ (Kaken Pharmaceutical, Tokyo, Japan) [10, 11]. Japanese sales of Seprafilm^®^ in FY2014 exceeded about 95 million US dollars (Kaken Pharmaceutical). Various studies are addressing the great need for materials to prevent postoperative pleural adhesions, but no product has yet been brought to market.

This study investigated the effectiveness of a novel adhesion-preventing membrane that utilizes surface water induction technology to prevent adhesions following thoracic surgery. In a previous study utilizing this principle, Noishiki and Shintani reported that this membrane is more effective than Seprafilm^®^ in preventing postthoracotomy pleural adhesions in dogs [12]. However, according to that study [12], although the membrane was highly effective in preventing adhesions in the immediate vicinity, severe adhesions formed at other locations. The present study employed hyaluronic acid, which has a long history as a highly hydrated and safe pharmaceutical, instead of the glycerin used by Noishiki and Shintani [12].

Our novel adhesion-preventing membrane is stored under refrigeration in the form of a dried sheet. After immersion in physiological saline, the membrane can be simply placed at the desired location after thoracotomy, and does not require any special reagents or equipment for either storage or use.

Recent studies have used aldehyde dextran and ε-poly (L-lysine) powder (D-L powder) [13], cross-linked poly(gamma-glutamic acid) powder (XL powder) [14], and Prevadh^®^ polyethylene glycol-containing film [15] as materials for preventing postoperative pleural adhesions. D-L powder successfully reduced the length of adhesions compared with a control group, but did not prevent them altogether. Similarly, although XL powder reduced adhesion scores between the lungs and pleura, adhesions still occurred. Prevadh^®^ polyethylene glycol-containing film has been found to be highly effective in preventing adhesions, but these results were obtained from the small pleural cavities of rats, and no data are available regarding efficacy in larger animals. Seprafilm^®^ has been found to exert significant anti-adhesion effects in pediatric cardiac surgery [16] and during thoracotomy in rats [17], but has been shown to be less effective than surface water induction technology in preventing postoperative pleural adhesions [12]. None of the anti-adhesive materials developed to date have successfully prevented the formation of postoperative pleural adhesions or have been brought into actual clinical use.

This study reports the implantation of our novel anti-adhesion membrane during thoracotomy in dogs. After two weeks, adhesions were evaluated by macroscopic and histopathological examinations. We also verified the ease of membrane handling during surgery.

## Materials and methods

Experimental operations were carried out in accordance with the Regulations on Animal Experiments of Tokyo University of Agriculture and Technology and with the Guide for the Care and Use of Laboratory Animals Eighth Edition (Committee for the Update of the Guide for the Care and Use of Laboratory Animals; National Research Council). All operations were approved by the Animal Experiments Subcommittee of Tokyo University of Agriculture and Technology (Permit number 27–36; April 1, 2007).

The experimental substance used was an insoluble hyaluronic acid anti-adhesion membrane comprising sodium hyaluronate and concentrated glycerin. The membrane of hyaluronic acid sodium was created from an aqueous solution of sodium hyaluronate. Some of the sodium hyaluronate changed to hyaluronic acid and formed hydrogen bonds, but was subsequently insolubilized and then neutralized. The membrane comprised 90% sodium hyaluronate and hyaluronic acid, and 10% glycerin in a dry state. When the dry membrane is dipped in saline, about the same weight of water will be absorbed. In terms of the external appearance and properties, the membrane comes as a colorless, clear, liquid-containing sheet (13.0 cm × 10.5 cm × 0.1 cm).

The animals used were eight beagles (TOYO Beagles; KITAYAMA LABES, Nagano, Japan), divided into an experimental group (five males) implanted with the novel anti-adhesion membrane, and a control group (three females). In the experimental group, adhesions were evaluated at a total of ten sites in the right and left pleural cavities of the five dogs. In the control group, adhesions were evaluated at a total of three sites in the left pleural cavities of the three dogs.

**Fig 1:**
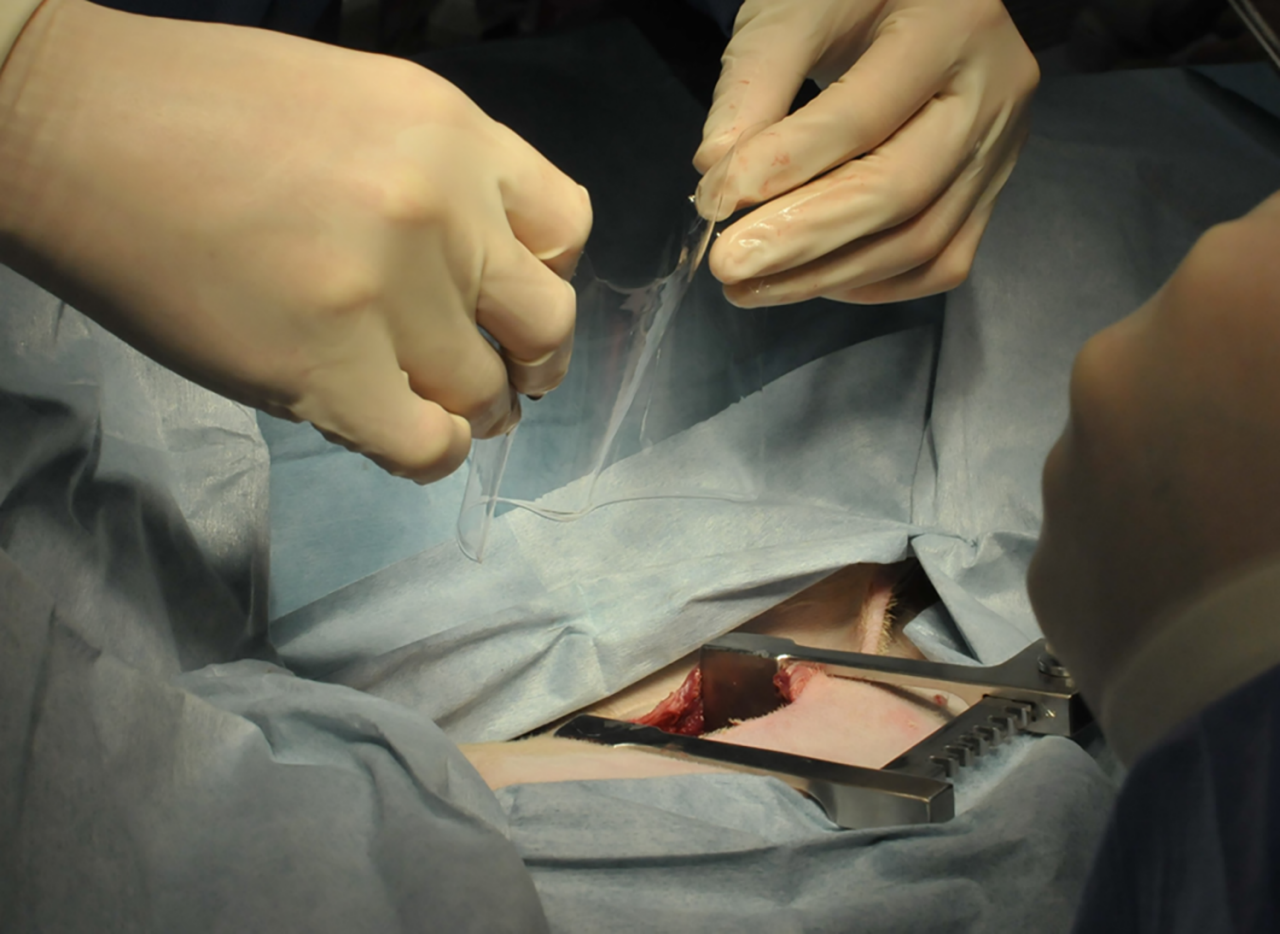
Insertion of the membrane into the pleural cavity. After exposure of the pleural surface to air, the anti-adhesion membrane, which had been immersed in physiological saline for approximately 5 min, was placed between the visceral and parietal pleura.

Before implantation, dogs were administered intravenous ampicillin sodium at 30 mg/kg (ampicillin Na injection; Kyoritsu Seiyaku, Tokyo, Japan) to prevent infection, and meloxicam (Metacam 0.5% injection solution; Boehringer-Ingelheim Vetmedica Japan, Tokyo, Japan) for pain relief. Following pre-administration subcutaneous injection of atropine sulfate at 0.02 mg/kg, butorphanol tartrate (Vetorphale^®^ 0.2 mg/kg iv; Meiji Seika, Tokyo, Japan), and midazolam (Midazolam Sandoz (injection solution) 0.2 mg/kg iv; Sandoz, Tokyo, Japan), general anesthesia was induced using propofol (Propofol Mylan 6 mg/kg iv; Mylan, Tokyo, Japan). After tracheal the intubation, inhalation anesthesia was maintained using isoflurane (Isoflurane for Animal Use, 1.0–2.0%; Intervet, Osaka, Japan). The implantation sites were the left and right pleural cavities of dogs in the experimental group. Approximately 15 cm of the 5th intercostal space was opened through the muscles overlying the pleural cavity (latissimus dorsi, serratus ventralis thoracis, intercostales externi, and intercostales interni muscles). After thoracotomy was performed by the usual method, the lung surface was exposed to the air for approximately 30 min to dry out the visceral pleura on the lung surface and thus create a mild level of invasiveness. After this exposure, an anti-adhesion membrane that had been immersed in physiological saline for about 5 min was placed between the visceral and parietal pleura (Fig 1), and a drainage tube was inserted. Half of the samples in each dog from the experimental group were fixed to the chest wall using tissue adhesive (Hydrofit; Terumo, Tokyo, Japan). Chest closure was performed with 2–0 synthetic absorbable sutures (polyglycomer monofilament, Byosin; COVIDIEN Japan, Tokyo, Japan) by the normal method after intercostal nerve block with bupivacaine. We installed a chest tube (internal diameter, 2.5 mm; outer diameter, 4.0 mm) in the pleural cavity. The control group underwent the same procedure for left-side chest closure, but without implantation of the anti-adhesion membrane. We performed suction by hand using a 10-mL syringe (every 2–3 h for the first 12 h, then every 12 h), with no sustained mechanical aspiration. The chest tube was removed 24–48 h postthoracotomy after confirming that no pleural fluid was being drained. For lung training, we let the dogs walk freely as soon as possible to optimize cardiac and pulmonary functions. Ampicillin sodium (1.5 mg/kg iv) was administered 1 week postoperatively to prevent infection. No agents that might exert anti-adhesive activity were used. Dogs were sacrificed 2 weeks postoperatively by intravenous administration of an overdose of potassium chloride (KCL Injection 20 mEq Kit; Terumo) under general anesthesia that was maintained with 5% isoflurane (Isoflurane for Animal Use; Intervet, Tokyo, Japan). The 6th or 7th intercostal space (i.e., one rib caudal to the initial surgery) was opened to macroscopically evaluate the severity of adhesions. Both the experimental membrane and surrounding tissue were examined both macroscopically and microscopically, and the following three evaluation items were observed and recorded: capsule formation or discoloration in surrounding tissues, discoloration or degeneration of the experimental membrane itself, and any other abnormalities. The presence and severity of adhesions was determined and scored according to the following scale: 0, no need for dissection; 1, membranous adhesion, membrane surface easily dissected; 2, slight adhesion, surface of adhesion could be dissected; 3, moderate adhesion, surface of adhesion difficult to dissect; 4, severe adhesion, surface of adhesion impossible to dissect.

Tissues containing parietal and visceral pleura were collected for histopathological examination and fixed in 10% neutral-buffered formalin solution. After fixation, the parts of the parietal pleura and lung lobes that contained the experimental membrane were resected and the tissues embedded in paraffin blocks, from which hematoxylin and eosin-stained specimens were prepared.

## Results

### Implantation of anti-adhesion membrane

**Fig 2:**
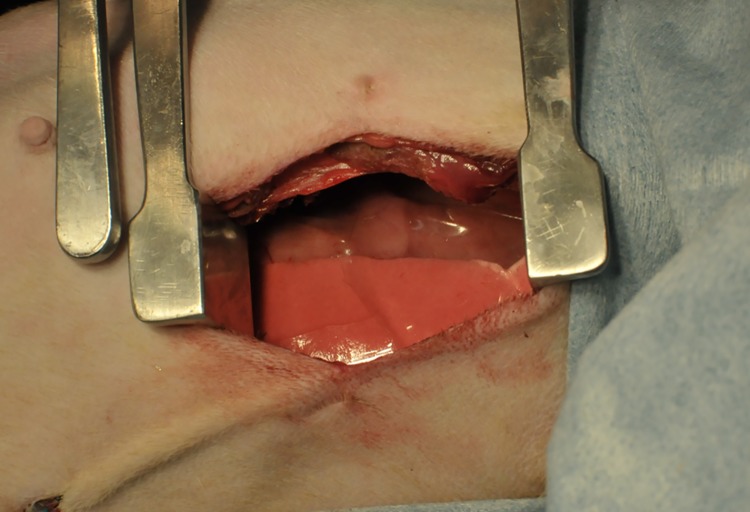
After insert the membrane in the pleural cavity. The anti-adhesion membrane was immersed in physiological saline (for approximately 5 min) prior to the thoracotomy. As a result, the membrane was sufficiently soft by the time chest closure was performed. The membrane did not break under normal handling.

The anti-adhesion membrane had been immersed in physiological saline prior to the thoracotomy and was thus sufficiently soft by the time chest closure was performed. The membrane did not break under normal handling (Fig 2), but could be damaged with forceps or other hard instruments. Insertion into the pleural cavity was comparatively easy. After insertion, the membrane was fitted onto the lung surface, and did not easily slip.

Tissue adhesive was spread onto the tip of a small probe and applied to the chest wall, after which respiration was maintained at approximately 10-s intervals with a respirator, and the anti-adhesive membrane adhered tightly to the chest wall.

#### Control group

**Fig 3:**
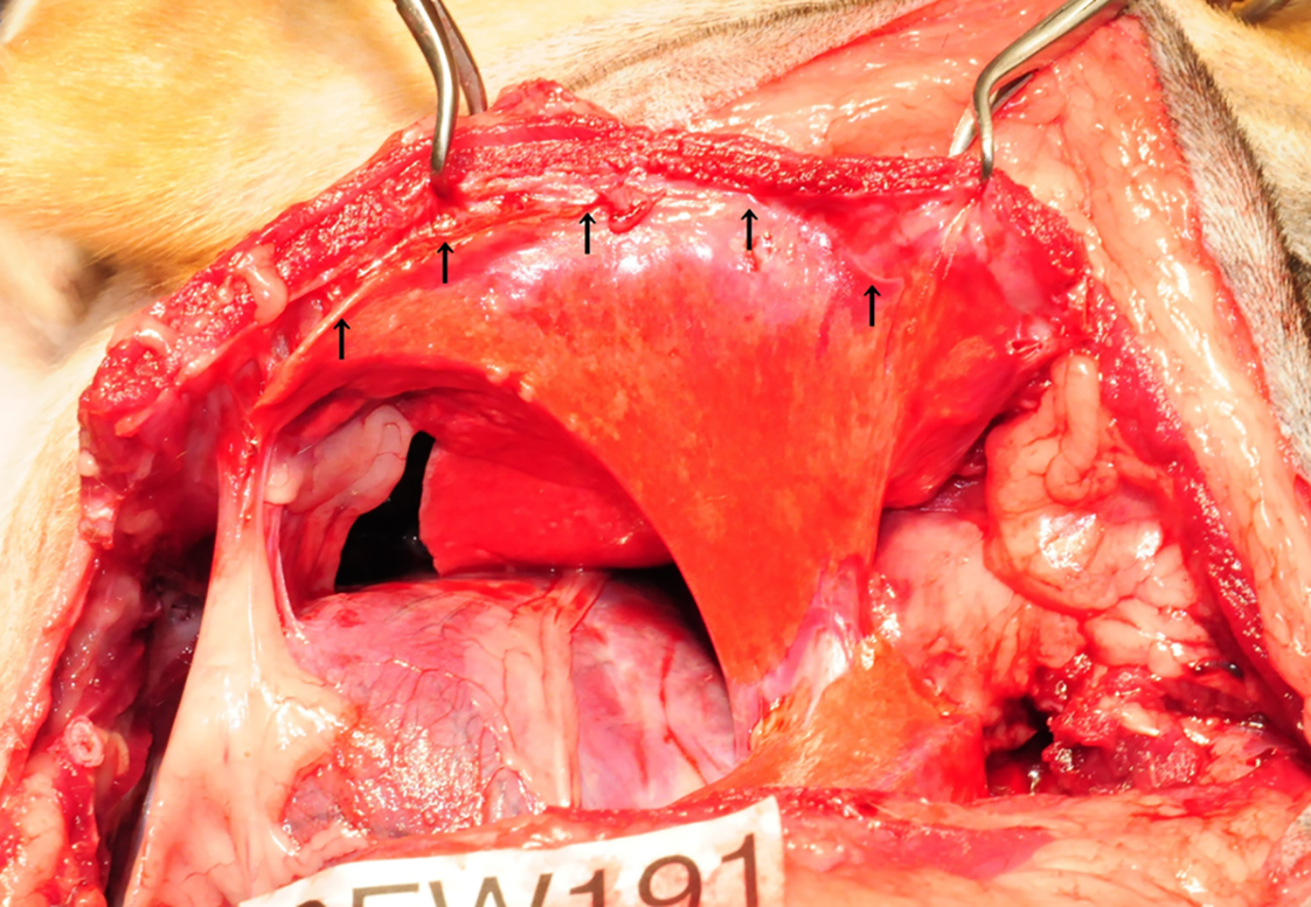
Views of adhesions between the parietal and visceral pleura in the control group. Severe adhesions between the parietal and visceral pleura are present at all three sites in the control group, mainly at the level of the 5th and 6th intercostal spaces.

Severe adhesions between the parietal and visceral pleura were present at all three sites in the control group (3/3) (median adhesion score, 4; N1: 4; N2: 4; N3: 3), mainly in the 5th and 6th intercostal spaces (Fig 3) (S1 Fig). These adhesions were strong, and blunt dissection was difficult. Adhesions were particularly severe with the chest wall sutures, and required sharp dissection.

#### Experimental group

**Fig 4:**
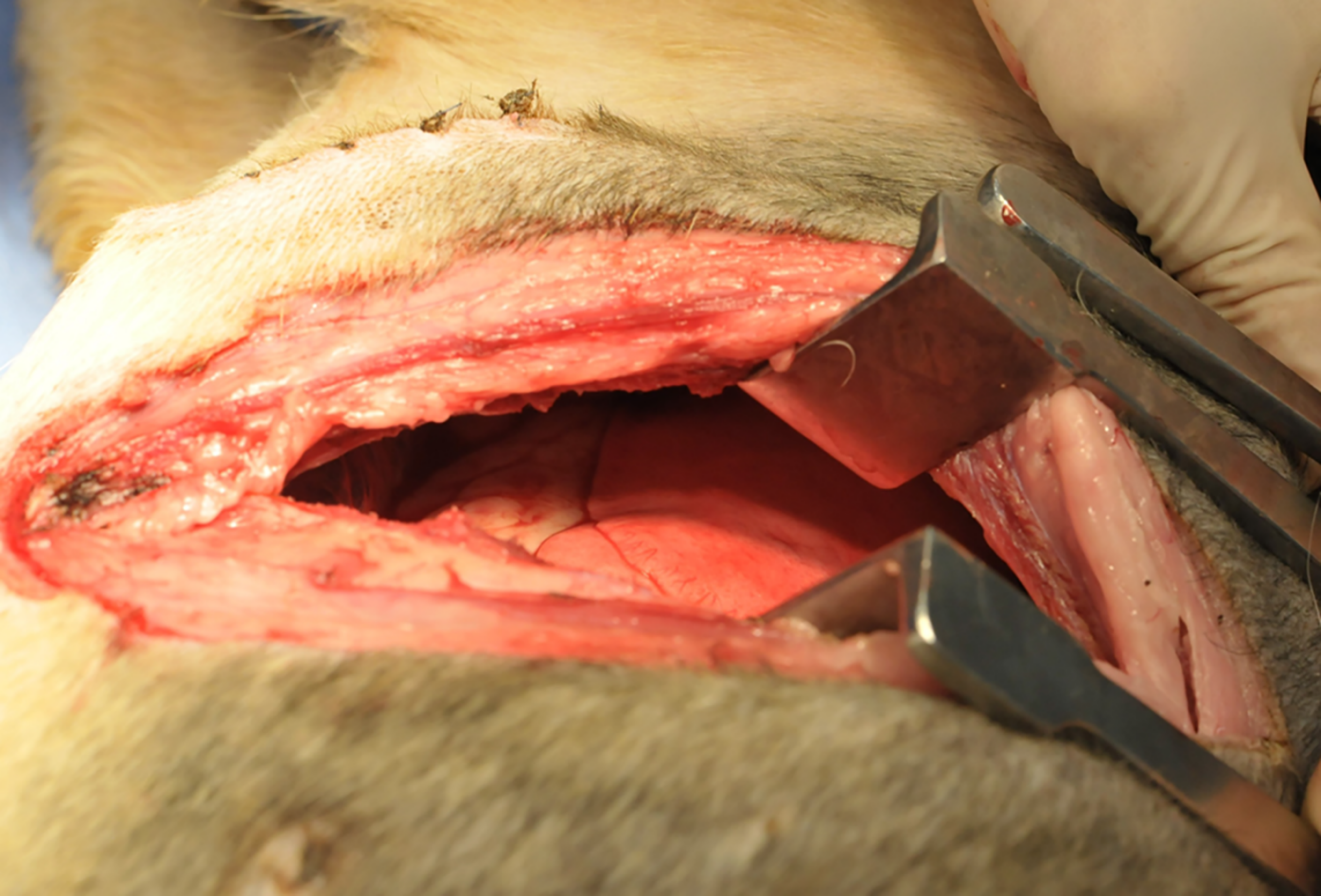
Views showing the lack of adhesions between the parietal and visceral pleura in the experimental group. No adhesions are evident between the parietal and visceral pleura in the experimental group. The pleura and lung lobe surfaces at the site of original thoracotomy are smooth, and no signs of inflammation are evident macroscopically.

Measurement of the adhesion between the parietal and visceral pleura revealed approximately 3 mm of attachment at one site in one dog (1 of 10 sites) (D5, left), and this attachment was easily dissected and did not pose a clinical problem (median adhesion score, 0). Pleural and lung lobe surfaces at the site of the original thoracotomy were smooth, with no signs of inflammation evident macroscopically (Fig 4) (S2 Fig). The use of tissue adhesive made no clear difference to the occurrence of adhesions, which were present at one of the five sites at which tissue adhesive had been used and none of the five sites at which no tissue adhesive had been used.

### Histopathological evaluation

#### Control group

**Fig 5:**
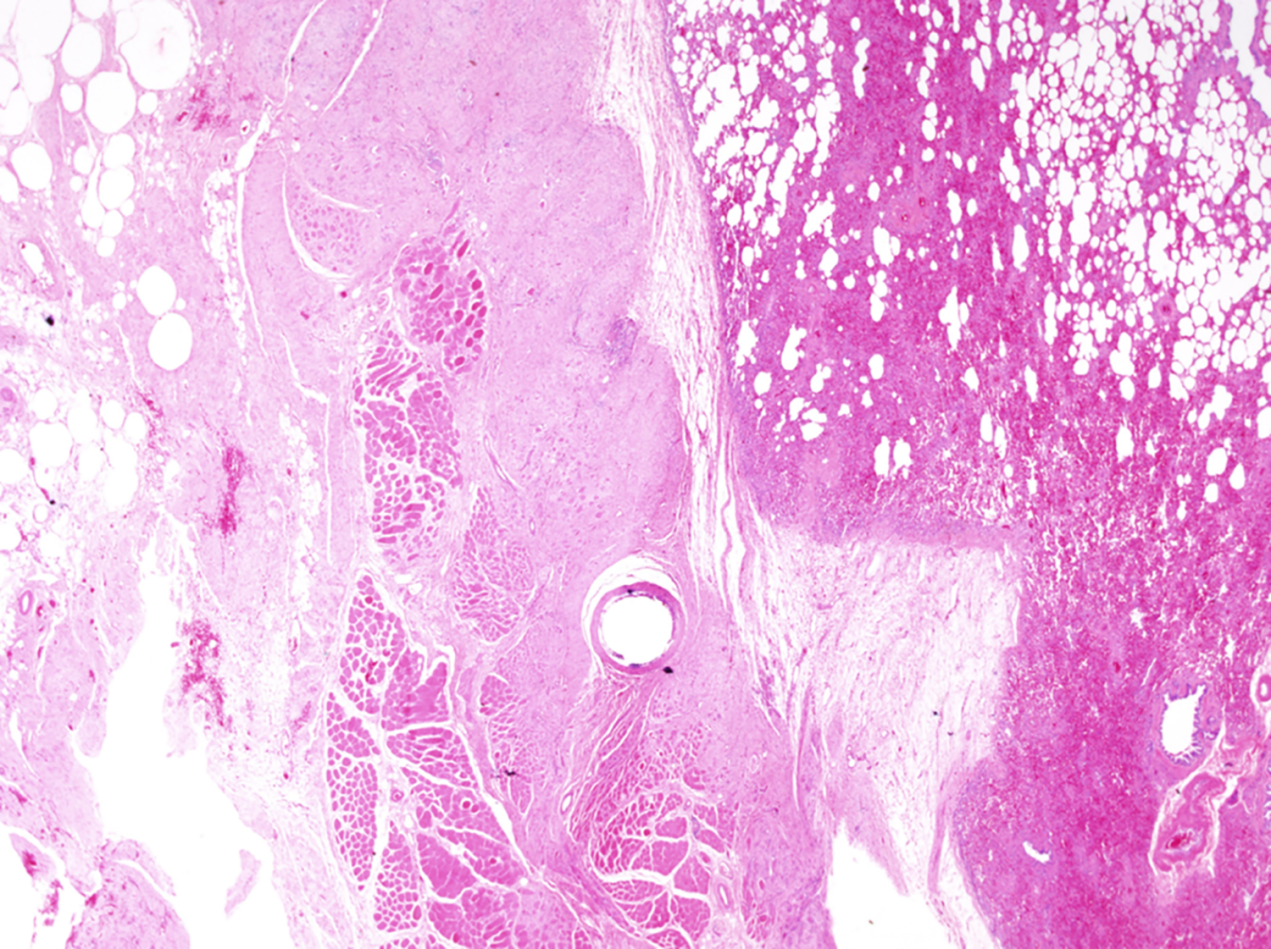
Histopathological observation in the control group (hematoxylin and eosin stain, × 12.5). Pronounced adhesions associated with severe fibrosis between the lungs and chest wall are present at all sites in the control group, with fibrosis extending to the muscle tissue of the chest wall.

Pronounced adhesions associated with severe fibrosis between the lungs and chest wall were present at all sites (3/3) in the control group, with fibrosis extending to the muscle tissue of the chest wall (Fig 5). In some locations, adhesions were associated with severe fibrosis extending into the muscle layer of the chest wall, granulomatous inflammation extending into the superficial layer of the lung parenchyma, and mild to moderate inflammatory cell infiltration, mainly by lymphocytes.

#### Experimental group

**Fig 6:**
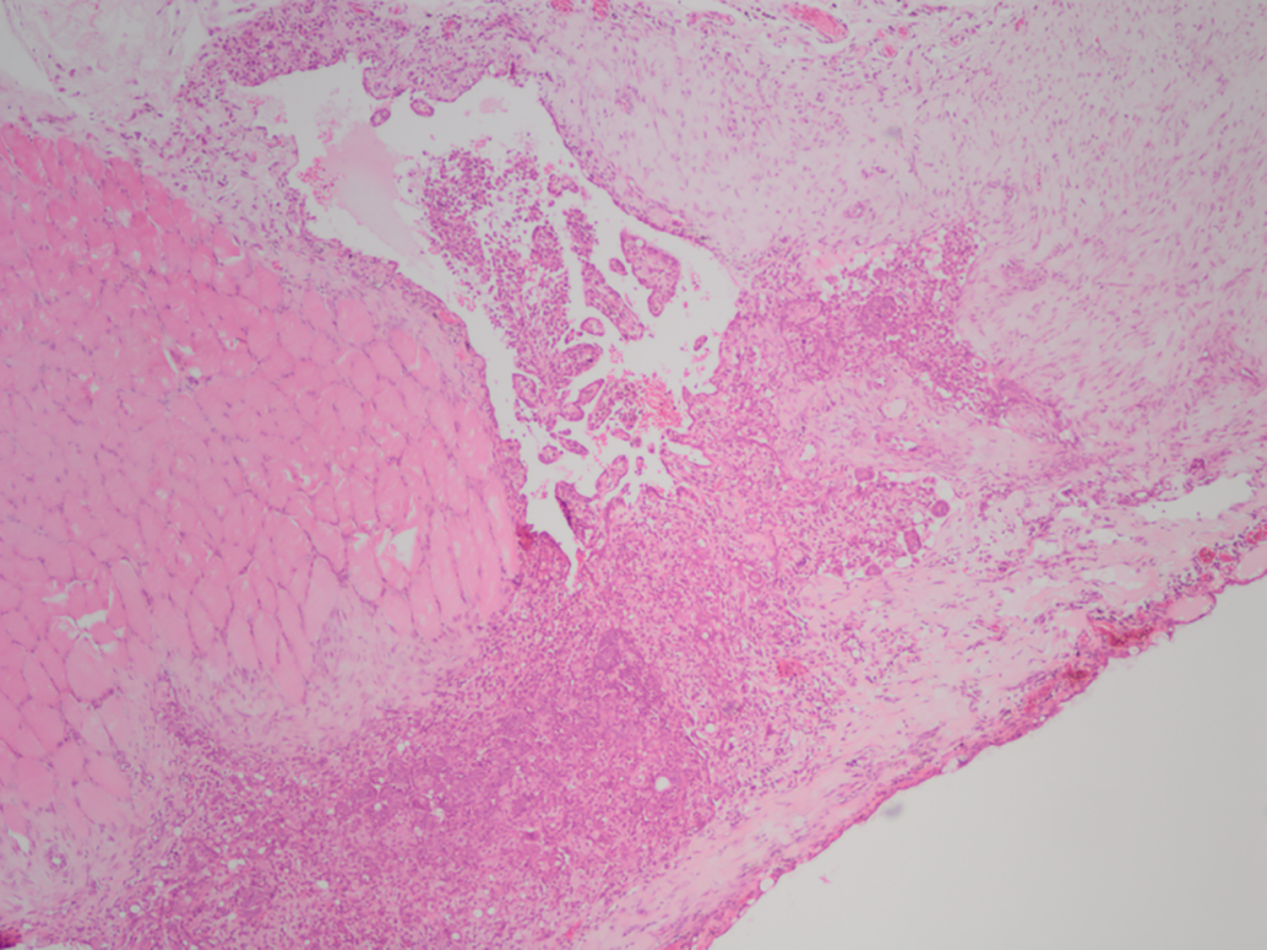
Histopathological observation in the experimental group (hematoxylin and eosin stain × 40). Villous proliferation is apparent in the pleura and connective tissue below the pulmonary pleura, but the severity of adhesions is scored as mild.

Although adhesions associated with severe fibrosis were observed at one site in one dog (between the D5 left pulmonary and parietal pleura), these changes were limited to only some of the specimens observed, and the severity of adhesions was scored as extremely mild. This was the only site at which adhesions were evident, and our study also histopathologically demonstrated the anti-adhesive effect of our membrane. In terms of notable changes other than adhesions, villous proliferation was apparent in the pleura and the connective tissue below the pulmonary pleura at all sites. Infiltration of inflammatory cells such as neutrophils and lymphocytes, hemorrhage, vascular proliferation, and fibrosis were all present in the stroma that had proliferated (Fig 6). In two dogs (bilateral pulmonary pleura in D1 and left pulmonary pleura in D5), a crusted hyaline film covered the surface below the media, blocking the spread of granulation tissue and fibrosis.

Necrosis of the treated site, inflammatory reaction, granulation tissue progressing to fibrosis, hemosiderin deposition in the lungs, fibrosis, and pneumonia were also seen. However, as an adhesion-inhibiting effect was evident and changes varied between individuals, these findings were regarded as secondary changes due to treatment. Scattered macrophage aggregation as a biological foreign body reaction and a foreign body response by multinucleated giant cells were seen in two dogs.

## Discussion

The use of our novel anti-adhesion membrane reduced the incidence of postthoracotomy pleural adhesions to an extremely low level, and adhesions were slight when they occurred. This anti-adhesion effect was confirmed histopathologically and the foreign body response was also very slight.

A number of studies have been conducted on materials to prevent postoperative pleural adhesions. D-L powder [13] and XL powder [14] have been shown to reduce the length and severity of adhesions, but adhesions still appeared in almost all cases. Prevadh^®^, a polyethylene glycol-containing membrane, successfully prevented the formation of adhesions, but pieces of experimental membrane measuring 3 cm × 3 cm were inserted into the small pleural cavities of rats, meaning that they covered most of the pleural cavity [15]. Seprafilm^®^, which is marketed as a membrane for preventing abdominal adhesions, has proven useful for preventing postoperative adhesions in pediatric heart surgery [16], and another study found a significant effect from Seprafilm^®^ in preventing postoperative mediastinal adhesions after mediastinoscopy in rats [17]. However, this approach has been shown to be less effective than surface water induction technology in preventing postoperative pleural adhesions [12].

In the present study, all individuals (3/3) in the control group developed adhesions severe enough to make sharp dissection unavoidable. In contrast, only one minor adhesion was seen at only one site in one dog (1/10) in the experimental group. Likewise, no macroscopic signs of inflammation were evident. Histopathological evaluation showed that severe adhesions, foreign body response, and inflammatory reaction were present in the control group, whereas only slight adhesion was seen in the experimental group. The foreign body response was also slight in the experimental group. The lower foreign body response observed in the experimental group compared to the control group seems paradoxical. We attributed the foreign body response in the control group to the thoracotomy, because a previous report described foreign body responses to suture thread and surgical gloves in postoperative adhesions [7]. As our examination was histopathological, we were unable to confirm clear causes of foreign body responses such as suture thread granuloma in the control group in this study. Notably, the chief constituent of the membrane used in the present study is sodium hyaluronate. Because hyaluronic acid is biocompatible in terms of biodegradability, the risk of secondary harmful phenomena is considered low [18]. Because inflammation is a strong vital reaction and did not appear while the membrane was in physical contact with the visceral and parietal pleura, and because adhesions after surgery were prevented by the minimal immune responses to the membrane, we think that the apparent paradox is explained.

In conclusion, our membrane appeared extremely effective in preventing adhesions. This method may reduce both the incidence and severity of adhesions. Adhesions that are easily dissected have no clinical significance. The membrane was designed to be used immediately after preoperative immersion in physiological saline, and no special equipment or reagents are required for storage or use.

As previously reported by Noishiki and Shintani [12], anti-adhesion membranes that utilize surface water induction technology appear highly effective in preventing adhesions and, in the pleural cavity, offer better efficacy than Seprafilm^®^, which was designed to prevent abdominal adhesions. We used hyaluronic acid as the main ingredient of our membrane. The water layer generated by hyaluronic acid, which promotes an extremely high water content, means the activity extends over a wider area than a membrane consisting of glycerin alone, as used by Noishiki and Shintani, and this may have been responsible for the positive results in our study.

Our study had two principal limitations. The first concerned the level of invasiveness. In this study, the lung surface was exposed to air for approximately 30 min in a mildly invasive procedure. In a real-life thoracotomy, hemorrhage and other complications may occur, increasing the level of invasiveness. Further studies are required to investigate the anti-adhesion effects of our membrane in highly invasive procedures with associated hemorrhage, such as pulmonary lobectomy. We plan to inspect the prevention of adhesion effects after chest surgery by the membrane in future investigations by increasing the surgical invasiveness using procedures such as lobectomy. The prevention of adhesions is important, both to reduce the burden on surgeons when reopening the chest in further chest surgeries and to reduce pulmonary burdens on patients after the initial surgery. According to previous reports, adhesions finish forming within 7 days after abdominal surgery, and do not form afterwards [19–21]. On the other hand, pleurisy from infection following lobectomy as a chest surgery may occur more than two weeks after thoracic surgery. The observation period of two weeks is shorter than the general period for re-thoracotomy in humans. The level of surgical invasiveness in this study was slight, and we considered that the risk of pleurisy was not increased. However, extension of the observation period will be necessary in the future for studies investigating more invasive procedures such as lobectomy for clinical application of this membrane. The second limitation relates to the use of video-assisted thoracic surgery (VATS). In recent years, chest surgery has been shifting away from thoracotomy toward less-invasive procedures using VATS. In many cases, VATS is performed using a port inserted through a small incision. How this membrane might be used with VATS, including the shape and method of use, is a subject for further study. Should the membrane prove feasible for use with VATS, a groundbreaking means of further reducing the invasiveness of chest surgery may be afforded.

In addition, the issue of bias between the experimental and control groups due to the differing sexes of the animals must be considered. This difference was due to the limitations of the sample supply and issues with obtaining ethics approval. Although a small number of reports have examined sexual influences on postoperative adhesion in humans, Barmparas et al. [22] subsequently reported that the evidence regarding a role of sex in adhesion formation after laparotomy remains insufficient. Based on these reports, we consider that the results of this study were unlikely to have been markedly influenced by differences in sex as a source of biological variability.

In terms of the clinical implications of our membrane, the following two advantages can be anticipated. The first is a high level of effectiveness in preventing postthoracotomy adhesions in the chest. Postoperative pleural adhesions are associated with a range of adverse effects. Not only do they cause complications during re-thoracotomy, but they also result in reduced respiratory function in one-off surgery. Adhesion may be a cause of decreased respiratory function after thoracic surgery [1]. An extremely large number of chest surgeries are performed every year, and materials that can effectively prevent postoperative pleural adhesions are sorely needed. Our novel anti-adhesion membrane does not permit the formation of most adhesions after thoracotomy, and may also ensure that those few that do form remain weak. The second advantage is that the foreign body response to our membrane appears very slight. However great the anti-adhesion effect, if the foreign body response at the body contact surface is severe, not only will the performance of a product not live up to its full potential, but it will also be unsuitable for clinical use from a safety perspective. The response to our membrane was only slight, suggesting great potential for clinical use.

## Conclusions

Our novel anti-adhesive membrane, which utilizes surface water induction technology, was highly effective in preventing postthoracotomy pleural adhesions. Anti-adhesion effects were also confirmed histopathologically, and foreign body responses were slight. The membrane can be used without the need to prepare any special reagents or equipment, and is also very easy to handle during surgery. Our results suggest that this membrane may offer an extremely effective material for preventing postthoracotomy pleural adhesions.

## Supporting information

S1 Fig Severe adhesions between the parietal and visceral pleura. Severe adhesions between the parietal and visceral pleura were present at all three sites in the control group (3/3). A: N1 (Adhesion Score (AS); 4), B: N2 (AS; 4), C: N3 (AS; 3).
(TIF)

S2 Fig Measurement of the adhesion between the parietal and visceral pleura. Measurement of the adhesion between the parietal and visceral pleura revealed approximately 3 mm of attachment at one site in one dog(1/10) (D5, left) (arrow). (experimental group) A: D1 Left side (Adhesion Score (AS); 0), B: D2 Left side (AS; 0), C: D3 Left side (AS; 0), D: D4 Left side (AS; 0), E: D5 Left side (AS; 1), F: D1 Right side (AS; 0), G: D2 Right side (AS; 0), H: D3 Right side (AS; 0), I: D4 Right side (AS; 0), J: D5 Right side (AS; 0).
(TIF)
