# Supplementary figures and images for: Effective new membrane for preventing postthoracotomy pleural adhesion by surface water induction technology
Source: PLoS One. 2017 Jun 27;12(6):e0179815. doi: 10.1371/journal.pone.0179815 (PMC5487045; doi:10.1371/journal.pone.0179815)

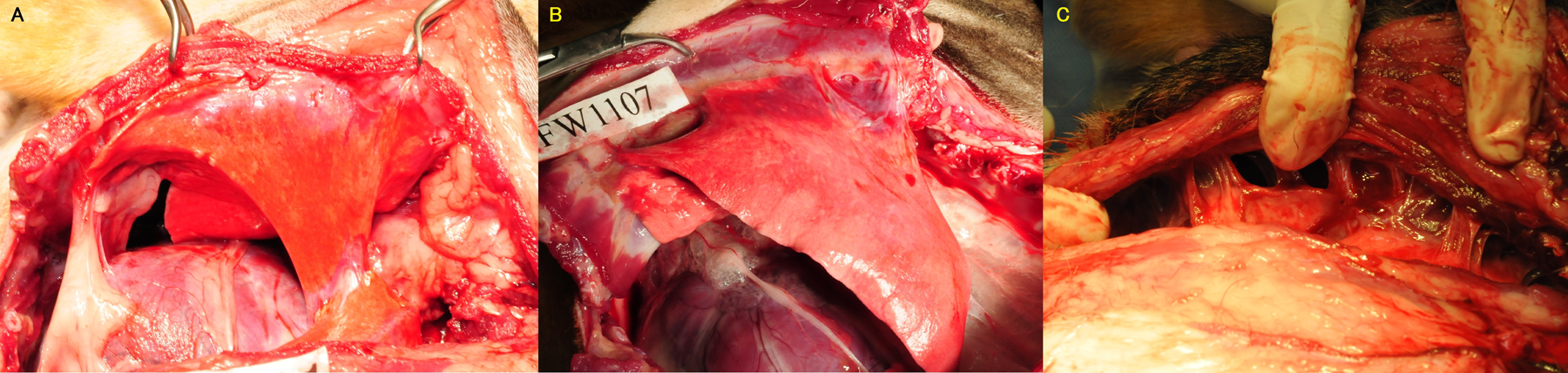

Supplement: S1 Fig — Severe adhesions between the parietal and visceral pleura were present at all three sites in the control group (3/3). A: N1 (Adhesion Score (AS); 4), B: N2 (AS; 4), C: N3 (AS; 3). (TIF) [file pone.0179815.s001.tif]

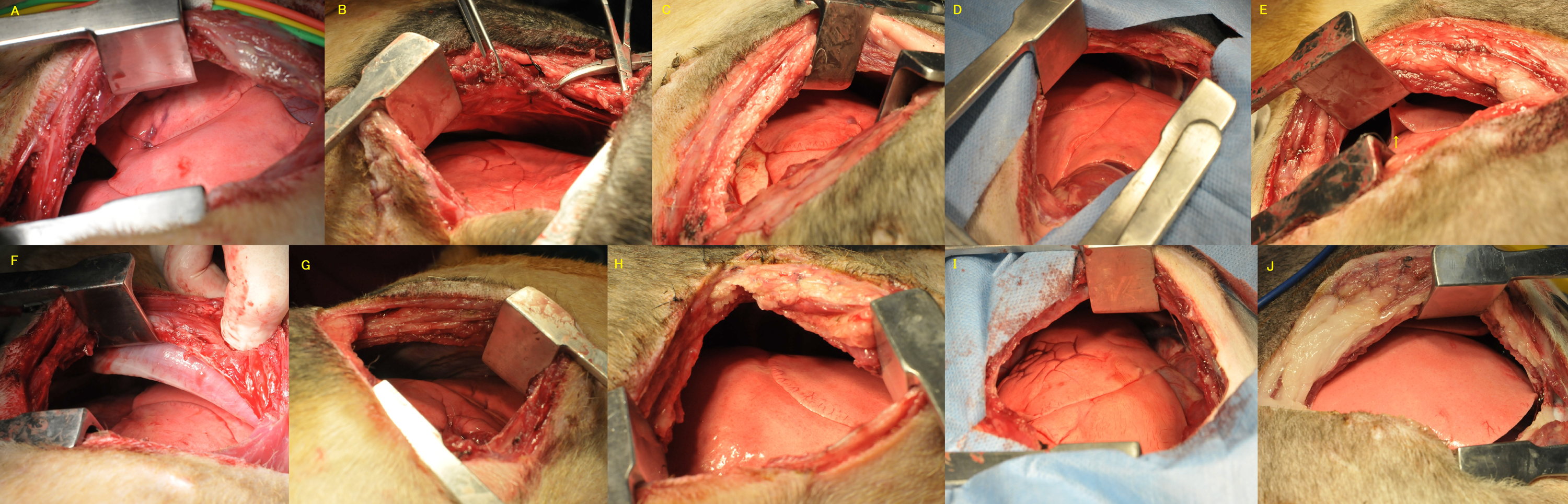

Supplement: S2 Fig — Measurement of the adhesion between the parietal and visceral pleura revealed approximately 3 mm of attachment at one site in one dog(1/10) (D5, left) (arrow). (experimental group) A: D1 Left side (Adhesion Score (AS); 0), B: D2 Left side (AS; 0), C: D3 Left side (AS; 0), D: D4 Left side (AS; 0), E: D5 Left side (AS; 1), F: D1 Right side (AS; 0), G: D2 Right side (AS; 0), H: D3 Right side (AS; 0), I: D4 Right side (AS; 0), J: D5 Right side (AS; 0). (TIF) [file pone.0179815.s002.tif]
